# Analyzing Workers’ Compensation Claims and Payments Made Using Data from a Large Insurance Provider

**DOI:** 10.3390/ijerph17197157

**Published:** 2020-09-30

**Authors:** Navneet Kaur Baidwan, Nathan W. Carroll, Bunyamin Ozaydin, Neeraj Puro

**Affiliations:** 1 Department of Health Administration, School of Health Professions, University of Alabama at Birmingham, Birmingham, AL 35294, USA; natcar@uab.edu (N.W.C.); bozaydin@uab.edu (B.O.); 2 Health Administration Programs, School of Business, Florida Atlantic University, Boca Raton, FL 33431, USA; npuro@fau.edu

**Keywords:** Workers’ Compensation, injuries, payments

## Abstract

Background: All states in the USA have established Workers’ Compensation (WC) insurance systems/programs. WC systems address key occupational safety and health concerns. This effort uses data from a large insurance provider for the years 2011–2018 to provide estimates for WC payments, stratified by the claim severity, i.e., medical only, and indemnity. Methods: Besides providing descriptive statistics, we used generalized estimating equations to analyze the association between the key injury characteristics (nature, source, and body part injured) and total WC payments made. We also provide the overall cost burden for the former. Results: Out of the total 151,959 closed claims, 83% were medical only. The mean overall WC payment per claim for the claims that resulted in a payment was $1477 (SD: $7221). Adjusted models showed that mean payments vary by claim severity. For example, among medical only claims, the mean payment was the highest for amputations ($3849; CI: $1396, $10,608), and among disability and death related claims, ruptures cost the most ($14,285; $7772, $26,255). With frequencies taken into account, the overall cost burden was however the highest for strains. Conclusions: Workplace interventions should prioritize both the costs of claims on average and the frequency.

## 1. Introduction

Occupational injury surveillance data are crucial to designing effective preventive interventions [1]. Currently, there exists no single, comprehensive national injury surveillance database, especially for nonfatal injuries. However, there are several databases that provide an opportunity for state or federal level injury surveillance. While some of these may only be demographic in nature, other contain information regarding related health outcomes and potential risk factors, such as workers compensation (WC) datasets. The latter provide information that can be used to conduct workplace hazard evaluations and suggest preventive interventions [2].

All the states within the United States (USA) have established WC insurance systems/programs that provide income protection, medical treatment, and rehabilitation to workers who get injured or ill due to their work. WC coverage is provided to all or nearly all workers for whom such coverage is required and covers over 90% of the USA wage and salary workers [3]. Importantly, because of their design and performance, WC systems address a key concern for the National Institute for Occupational Safety and Health given the implications for occupational safety and health and post-injury outcomes [4].

Note that while there is no comprehensive national surveillance system for occupational injuries and illnesses, the WC data, despite their limitations, are recognized as one of the major data sources to address the former [1]. In fact, WC claims have now long been used to conduct occupational health and safety research and identify priority preventive interventions [3]. While the primary purpose of maintaining a record for WC claims is to ensure appropriate payment to the injured worker, these records still contain crucial public health data regarding nature and source of the injury, body part injured, injury severity, lost work time, and occupation [3]. Furthermore, the WC databases are recognized to have substantial utility for the purposes of epidemiological research given their high sensitivity for case ascertainment [5].

In the year 2007, WC programs covered around 132 million workers and paid over $55 billion in benefits across the USA including over $27 billion for medical care and over $28 billion as cash benefits (for lost work time). The latter differ by the duration and severity of the impairment or disability with temporary total disability benefits being paid when the workers cannot temporality perform pre-injury work. While, in case of total temporary disability, the workers receive two-thirds of their pre-injury wages (varies by state); in case of temporary partial disability where workers return to work before maximum medical improvement and perform reduced work tasks with lower pay, they receive temporary partial disability benefits. In other cases, if the disability is permanent in nature but the worker is not completely limited in their ability to perform tasks, they receive permanent partial disability benefits. On the other hand, permanent disability benefits are paid if the disability is considered permanent following maximum medical improvement [6].

There is still however a dearth of literature with regards to the severity of such injuries across specific injury characteristics and their relation with direct WC payments [7]. Further, existing literature that has used WC data to estimate associated payments has been limited in scope with regards to sample size, states included, limited injury characteristics analyzed individually, etc. [8,9,10,11]. This research effort uses data from a large insurance program covering several years and all fifty states to provide estimates for WC payments across specific injury characteristics related categories, stratified by the claim severity.

## 2. Materials and Methods

### 2.1. Study Sample

Data for this study were obtained from a large, national third-party provider for WC claims. Overall there were over 6 million claims including both work-related injuries and illnesses. Out of these, this effort focuses on 151,959 closed claims corresponding to work-related injuries from the years 2011–2018.

### 2.2. Variables

Claim type: This research effort focusses only on closed claims to ensure that our measures of cost are not truncated. Therefore, all other types of claims including those open or incomplete were excluded from this analysis. Key injury characteristics: These include National Council on Compensation Insurance (NCCI) based classifications for the nature of injury, source of injury, and body part injured. Further information on each of these individual categories is available elsewhere [12]. These are also provided in the tables ahead. Lastly, severity of injury was classified as medical only, and indemnity (disability including permanent disability (total and partial), temporary disability (total and partial), and death) related. Other variables: gender (male, female), marital status (married, never married, divorced, separated), age at injury (calculated as date of injury-date of birth), years of work experience until the date of injury (calculated as date of injury-date of hire), employment status (full time, part time including seasonal employees), and state in which the injury occurred. Total WC payment: These were calculated as a sum of total indemnity and medical and expense payments made on a claim.

Please note that while job titles were available, these were not coded using a standard tool and were available as free text. Therefore, these could not be used for these analyses. Additionally, given the large proportion (>50%) of missing information for lost workday counts, the latter were not included either.

### 2.3. Analyses

We provide descriptive statistics (frequencies, and percentages) for each of the key injury characteristics stratified by the claim’s severity, i.e., medical, and indemnity (death and disability) claims. We used generalized estimating equations (GEE) [13] with a log link and gamma distribution and accounted for correlation within claim IDs nested within the insurance company’s clients to analyze the association between the key injury characteristics and WC payments made. To clarify, the standard equation for generalized linear models (GLMs) can be expressed as: Y = g (β0 + β1X1 + β2X2 + ... + βkXk + e), where “g” is the link function. In case of correlated outcomes, GEE can build upon for the GLMs to account for the former [13]. All these models were adjusted for age at injury, gender, years of work experience, employment status, and state (because of states having differing medical fee schedules and regulations about compensation for lost wages). These covariates were selected based on Directed Acyclic Graphs [14]. We also noted that the model fit improved on addition of these covariates as compared to the crude model. All these models were stratified by the severity of claim categorized as medical only and death and disability-related claims. Death and disability-related claims were analyzed together as indemnity related claims due to low cell counts for death-related claims (n = 24) across several categories within the three key injury characteristics. In addition to estimating mean payments using GEE models, as a supplement, we also provide estimates of the “burden” posed by each injury characteristic by multiplying its frequency in the data by its mean cost. The Supplementary Tables provide the top five most burdensome claims for each category [15].

## 3. Results

There were 272,476 total closed claims in the study period (2011–2018). Out of these, for 13,945 claims, the state in which the injury occurred was not provided. Furthermore, nature and source of injuries that either corresponded to an illness or included no physical injury were also excluded from these analyses (n = 33,575). Injury severity codes that were labelled as “accidents only” were also excluded (n = 41,667). The analyses were further restricted to workers with age at injury being up to 85 years and those older were excluded (n = 213). Next, those with unknown employment status were also excluded (n = 29,870). Lastly, since almost all of these claims were reported in the year 2011 or later, the analyses were further limited to only these years (n = 151,959).

Out of these, 126,614 were medical only, and 25,345 were disability and death-related claims. Among the latter, 88% were temporary, and 11% were permanent disabilities. There were 51% males (n = 77,236) in this study. Around 33% were in the age group of 30–45 and 45–60 years old, while around 23% were in the 18–30 years old group. Around 76% of the claims involved full-time workers; 30% of these had less than one year of work experience, and 24% had more than 10.

Further descriptive statistics showed that strains, followed by contusions and lacerations, were the most common nature of injuries (Table 1) accounting for respectively 30%, 21%, and 12% of the total claims. Moreover, strains, contusions, and lacerations combined accounted for 61% of death and disability and 63% of medical only claims.

Table 2 presents frequencies and percentages for the source of injury by the claim type. Overall, falls/slips were the most common source of injury, followed by strains and being struck by something or someone.

Table 3 shows that the most common body parts injured included wrist and hand (n = 33,187), accounting for 23% of the medical only and 14% of the disability and death related claims. These were followed by injuries involving multiple body parts (n = 16,685) and the low back area (n = 15,610).

Our data include claims payments totaling $190 million approximately resulting from 128,688 WC payable claims over the study period. Of these, 81.4% are associated with medical only claims while the rest are associated with disability and death claims. The mean overall WC payment per claim for the claims that resulted in a payment was $1477 (Standard Deviation (SD): $7221). The mean payment varied by the type of claim with claims related to death (n: 24) resulting in a mean payment of $25,389 (SD: $56,790). This was followed by permanent disability (n: 2642, mean payment: $15,493, SD: $34,183), temporary disability (n: 21,235, Mean payment: $3255, SD: $10,772), and medical only claims (n: 104,757, mean payment: $754, SD: $1930). Note that, a total of 1414 disability and death related claims and 21,857 medical only claims did not incur a WC payment. The estimates provided ahead are further provide multivariable adjusted mean payment estimates and claim type stratified GEE models for each key injury categories, i.e., nature and source of injuries and body part injured. The estimates are from GEE models accounting for correlations within claims nested within each client and adjusted for age at injury, gender, years of work experience, employment status, and state. Overall burden (Tables S1–S3) for each of the former stated categories was estimated using mean payment estimates and frequency of each claim. These estimates also highlight the key injury characteristics that are overall associated with the highest cost burden. We additionally identify five of the most common medical and indemnity-related claims.

Table 4 shows that adjusting for covariates mentioned earlier, among medical only claims, with concussions as the reference, the mean payment was the highest for amputations (mean payment: $3849; Confidence Interval (CI): $1396, $10,608). Among disability and death related claims, ruptures cost the most on average with a mean payment of $14,285 ($7772, $26,255). Overall, with reference to Concussions, the five most burdensome (calculated as a product of frequency of respective claims and mean payment) medical-related claims included Strains, Contusions, Lacerations, Sprains, and Punctures. Similarly, the five most burdensome disability and death-related claims included Strains, Sprains, Contusions, Fractures, and Multiple injuries (Table S1).

As can be seen in Table 5, with reference to burns and scalds from chemicals, the most expensive medical claims on average were motor vehicle collisions that resulted in a mean per claim payment amounting to $1089 (CI: $798, $1486). Among indemnity related, i.e., disability and death related claims, the mean payment was the highest for falls or slips from a ladder or scaffolding ($7136; $4950, $10,289). The respective burden, taking into account the frequency of each category of source of injury, is provided in Table S2. Table S2 shows that with reference to chemicals related burn or scalds, the five most burdensome medical related claims were Fall/Slip-On Ice or Snow, Strain/Injury By-Reaching, Motor Vehicle—Collision with other Vehicle, Cut/Puncture/Scrape—Object Being Lifted or Handled, and Fall/Slip—From Liquid or Grease Spills. The same for indemnity, i.e., disability and death related claims were Strain/Injury By—Lifting, Fall/Slip—On Same Level, Strain/Injury By— repetitive Motion–Carpal Tunnel Syndrome, Struck/Injured By—Another Person, and Strain/Injury By—Pushing or Pulling.

With reference to injuries to the lower log, injuries to body parts that were associated with high WC payments on average among medical only claims, besides those involving multiple body parts and other multiple injuries, including injuries to the spinal cord (mean payment: $911; $722, $1148). Next, among disability and death related claims, spinal cord injuries were the most expensive injuries with a mean payment of $11,115 (2144, 57,637) (Table 6). As far as overall burden of these injuries is concerned, as can be identified from the Table S3, the five most burdensome medical claims related injuries with respect to the reference were injuries to the wrist and hand, multiple body parts being injured, injuries to the low back area, ankle and feet, and knee. The same for disability and death related claims include multiple body parts being injured, injuries to low back area, wrist and hand, knee, and shoulder(s).

## 4. Discussion

There were an estimated 155 million workers in the USA civilian labor force in the year 2012. Around 3 million of those in private industry and over 800,000 in state and local government experienced a nonfatal injury or illness. Such injuries and illnesses are known to attribute to a cost burden of $200 billion annually [16]. Out of these, as per the most recent national estimates available from the year 2010, the WC insurance system covered over 124 million and involved a total cost burden of around $71 billion [17]. This research effort analyzed WC related payments incurred by a large third-party provider for the years 2011–2018. The 128,688 out of the total 151,959 closed claims for which a WC payment was made carried a burden of over $190 million. Around 81% (n = 104,757) of the claims were medical only, the majority of which were attributed to strains and contusions. The most common source of injuries were falls/slips in general. Finally, common body parts injured included wrist and hand, low back area, and ankle and feet.

A previous study suggested that it is important to estimate the WC payments since these can provide insights into the national trends for medical and other indemnity costs [18]. Another effort reported that temporary disabilities are the most common type of cash benefits, accounting for 63% of the cases with cash benefits and 17% of the total benefits incurred. On the other hand, while permanent total disabilities and fatalities account for 1% of the cases with cash benefits, they represent about 17% of the total cash benefit payments by the WC program [7]. In the current study, among the claims for which a WC payment was made, 81.4% were related to medical only claims, and the rest were death and disability related claims. While the former resulted in an overall WC payment of $111,129,376, the latter amounted to $78,988,043 in WC payments over the study period.

Next, we found that the most common body parts injured were wrist and hand, multiple body parts, and low back. Subsequently, with over $900 in mean WC payments, multiple injuries, including multiple head injuries were the most expensive claims on average. Spinal cord injuries, followed by shoulder injuries, were the most expensive death and disability claims based on the mean payments. Several previous research efforts focusing on particular states and specific occupations have used WC systems data to estimate the prevalence of different types of workplace injuries and the characteristics of affected workers. For example, a 10 year-long study [19] conducted among workers in the private ambulance services industry in Ohio found that around 54% of the claimants were males, and the majority (40%) were in the 25–34-year-old age-group. Around 60% of the claims were attributed to sprains and strains combined. Another previous effort [20] that analyzed data from over 232,000 unique claims from a large Maryland WC provider from 1998–2008 reported that the most common body parts injured were back, knee, and hand. The mean compensation was $6785 across all injury types. Multiple injuries accounted for around 64% of the total WC payments [20]. Another effort [21] that focused on agricultural injuries among workers in Colorado from 2000–2004 found an average cost of $7488 and $12,299 for vehicle-related medical and indemnity claims, respectively. As far as body parts injured are concerned, the most expensive medical and indemnity claims were respectively associated with leg and spine/back. Finally, dislocations were in general the most expensive claims.

### 4.1. Strengths

This study analyzed payments for both medical and indemnity claims across key injury characteristics across all fifty states, controlling for important confounding variables that may not be always available to researchers using these data, e.g., employment status, years of work experience, etc. Thus, the results of this study, given its wide scope, may be more representative of the USA workforce [18]. This study is also among the limited number of studies that have been able to analyze the payments across very specific injury characteristics. Our results provide information that could be useful in designing injury preventive interventions [20].

### 4.2. Limitations

The occupations among which the claimants were employed would have been an important variable to adjust for. Another limitation of using WC data is that nonfatal injuries and illnesses may be underreported in WC claims. However, this is also a limitation of other sources of data for work-related injuries and illnesses. In particular, the widely used Bureau of Labor Statistics (BLS) data have been reported to substantially underestimate nonfatal injuries and illnesses [22,23]. In fact, previous estimates reported by a study that linked individual cases between BLS and WC data in six states found that while WC systems missed over 180,000 lost-time injuries, BLS missed almost 340,000. Further, around 69,000 injuries were unreported by either of the aforementioned surveillance systems [22]. Another limitation relates to the true prevalence of strain-related injuries. These are one of the most common nature of injury codes reported, and yet, it is well known that this injury category is subject to potential misuse/classification error. While this limits the conclusions that injury epidemiologists can draw from our data, our results are nonetheless important to corporate risk managers seeking to understand costs imposed by employees reporting these injuries. Besides these, other limitations of the WC data itself include that no WC dataset covers the universe of employment injuries, nor does any WC dataset necessarily provide a representative sample. There are several reasons for this, including, underreporting of injuries to WC programs, WC data only covering claims from a particular WC provider company. In addition, costs derived from WC claims may underestimate the true extent of lost wages since workers are only compensated for a portion of their wages and because workplace injuries are underreported to WC companies. Finally, WC data can be challenging to access because they are protected for proprietary and privacy purposes by the companies that manage those claims. Besides, there exists no central repository for WC claims within the USA [1].

## 5. Conclusions

This effort uses data from a large third-party insurance provider and provides estimates for WC payments across specific categories of injury characteristics. Overall, the estimates show that injuries pose a huge economic burden to the WC system. Priority intervention may target specific injury characteristics that are associated with higher payments. However, it is also important to recognize that certain types of injuries like strains, which are associated with lower mean payments, are also the most frequent types of injuries. It is therefore also crucial to consider the frequencies of various injury causing mechanisms and body parts injured while designing workplace interventions.

## Figures and Tables

**Table 1:** Descriptive frequency and percentages for nature of injury categories, stratified by type of claim, medical only or death and disability related claims.

Nature of Injury	N (% of the Total Claims)		
	Disability or Death	Medical Only	Total
Amputation	15 (0.06)	13 (0.01)	28
Burn	450 (1.78)	4211 (3.33)	4661
Bite/sting	29 (0.11)	307 (0.24)	336
Concussion	209 (0.82)	673 (0.53)	882
Contusion	3591 (14.17)	28,060 (22.16)	31,651
Carpal tunnel syndrome	135 (0.53)	200 (0.16)	335
Crushing	265 (1.05)	1141 (0.90)	1406
Dislocation	102 (0.40)	223 (0.18)	325
All other specific injuries	2570 (10.14)	10,347 (8.17)	12,917
Foreign body	228 (0.90)	4038 (3.19)	4266
Fracture	785 (3.10)	1397 (1.10)	2182
Hearing loss (traumatic only)	28 (0.11)	72 (0.06)	100
Heat prostration	83 (0.33)	508 (0.40)	591
Irritation	22 (0.09)	82 (0.06)	104
Laceration	1457 (5.75)	16,462 (13.0)	17,919
Poisoning	38 (0.15)	263 (0.21)	301
Puncture	441 (1.74)	7922 (6.26)	8363
Rupture	60 (0.24)	95 (0.08)	155
Sprain	2972 (11.73)	10,477 (8.27)	13,449
Strain	10,433 (41.16)	35,242 (27.83)	45,675
Asphyxiation	8 (0.03)	32 (0.03)	40
Vision loss	22 (0.09)	79 (0.06)	101
Multiple injuries	888 (3.50)	3699 (2.92)	4587
All other cumulative injuries	398 (1.57)	681 (0.54)	1079
Unknown	116 (0.46)	390 (0.31)	506
Total	25,345	126,614	151,959

**Table 2:** Descriptive frequency and percentages for source of injury categories, stratified by type of claim, medical only or death and disability related claims.

Source of Injury	N (%)		
	Disability or Death	Medical Only	Total
Burn or Scald—Chemicals	60 (0.24)	524 (0.41)	584
Burn or Scald—Dust, Gases, Fumes or Vapors	51 (0.20)	429 (0.34)	480
Burn or Scald—Electrical Current	64 (0.25)	184 (0.15)	248
Burn or Scald—Fire or Flame	101 (0.40)	1050 (0.83)	1151
Burn or Scald—Hot Objects or Substances	6 (0.02)	101 (0.08)	107
Burn or Scald—Steam or Hot Fluids	54 (0.21)	712 (0.56)	766
Burn or Scald—Temperature Extremes	72 (0.28)	389 (0.31)	461
Burn or Scald—not otherwise classified	144 (0.57)	1781 (1.41)	1925
Caught In/Between—Machine or Machinery	139 (0.55)	1134 (0.90)	1273
Caught In/Between—Object Handled	349 (1.38)	2647 (2.09)	2996
Cut/Puncture/Scrape—Broken Glass	234 (0.92)	3078 (2.43)	3312
Cut/Puncture/Scrape—Hand Tool, Utensil Not Powered	110 (0.43)	698 (0.55)	808
Cut/Puncture/Scrape—Object Being Lifted or Handled	450 (1.78)	7002 (5.53)	7452
Cut/Puncture/Scrape—Powered Hand Tool, Appliance	551 (2.17)	2033 (1.61)	2584
Caught In/Between— not otherwise classified	90 (0.36)	816 (0.64)	906
Cut/Puncture/Scrape— not otherwise classified	277 (1.09)	3371 (2.66)	3648
Fall/Slip—From Different Level (Elevation)	235 (0.93)	1517 (1.20)	1752
Fall/Slip—From Ladder or Scaffolding	78 (0.31)	270 (0.21)	348
Fall/Slip—From Liquid or Grease Spills	1132 (4.47)	6476 (5.11)	7608
Fall/Slip—Into Openings	207 (0.82)	794 (0.63)	1001
Fall/Slip—On Ice or Snow	2030 (8.01)	9712 (7.67)	11,742
Fall/Slip—On Same Level	231 (0.91)	1629 (1.29)	1860
Fall/Slip—On Stairs	284 (1.12)	1268 (1.00)	1552
Fall/Slip— not otherwise classified	2237 (8.83)	10,506 (8.30)	12,743
Miscellaneous Causes—Absorption, Ingestion, Inhalation	187 (0.74)	1107 (0.87)	1294
Miscellaneous Causes—Foreign Matter in Eyes and Ears	196 (0.77)	3305 (2.61)	3501
Miscellaneous Causes	2709 (10.69)	6511 (5.14)	9220
Miscellaneous Causes—Person in Act of a Crime	322 (1.27)	1932 (1.53)	2254
Motor Vehicle—Collision with another Vehicle	2574 (10.16)	8330 (6.58)	10,904
Motor Vehicle—Collision with a Fixed Object	930 (3.67)	3267 (2.58)	4197
Motor Vehicle—Vehicle Upset	156 (0.62)	488 (0.39)	644
Motor Vehicle—Collision, not otherwise classified	393 (1.55)	1216 (0.96)	1609
Rubbed/Abraded—Repetitive Motion	30 (0.12)	107 (0.08)	137
Strain/Injury By—Holding or Carrying	180 (0.71)	1156 (0.91)	1336
Strain/Injury By—Jumping	271 (1.07)	2146 (1.69)	2417
Strain/Injury By—Lifting	344 (1.36)	2598 (2.05)	2942
Strain/Injury By—Pushing or Pulling	29 (0.11)	337 (0.27)	366
Strain/Injury By—Reaching	1109 (4.38)	8813 (6.96)	9922
Strain/Injury By—repetitive Motion–Carpal Tunnel Syndrome	621 (2.45)	5029 (3.97)	5650
Strain/Injury By—Twisting	236 (0.93)	563 (0.44)	799
Strain/Injury By—Using Tool or Machinery	60 (0.24)	241 (0.19)	301
Strain/Injury By— not otherwise classified	138 (0.54)	716 (0.57)	854
Striking/Stepping—Moving Parts of Machine	910 (3.59)	4356 (3.44)	5266
Striking/Stepping—Object Being Lifted or Handled	150 (0.59)	935 (0.74)	1085
Striking/Stepping—Stationary Object	646 (2.55)	4090 (3.23)	4736
Striking/Stepping—Stepping on Sharp Object	187 (0.74)	1107 (0.87)	1294
Striking/Stepping—Striking Against or Stepping on	21 (0.08)	194 (0.15)	215
Struck/Injured By—Another Person	338 (1.33)	3599 (2.84)	3937
Struck/Injury By—Animal or Insect	181 (0.71)	3166 (2.50)	3347
Struck/Injury By—Falling or Flying Object	2021 (7.97)	5553 (4.39)	7574
Struck/Injury By—Hand Tool or Machine in Use	322 (1.27)	1932 (1.53)	2254
Struck/injury By—Motor Vehicle	211 (0.83)	506 (0.40)	717
Struck/Injury By—Moving Parts of Machine	125 (0.49)	136 (0.11)	261
Struck/Injury By—Object Being Lifted or Handled	15 (0.06)	139 (0.11)	154
Struck/Injury By—Object Handled by Others	1049 (4.14)	2359 (1.86)	3408
Struck/Injury By— not otherwise classified	477 (1.88)	452 (0.36)	929
Total	25,345	126,614	151,959

**Table 3:** Descriptive frequency and percentages for body part injured, stratified by type of claim, medical only or death and disability related claims.

Body Parts Injured	N (%)		
	Disability or Death	Medical Only	Total
Missing	123 (0.49)	1616 (1.27)	1737
Abdomen including Groin	466 (1.84)	1574 (1.24)	2040
Ankle and Feet	2627 (10.36)	10,898 (8.61)	13,525
Brain	72 (0.28)	127 (0.10)	199
Buttocks and hip	318 (1.25)	1407 (1.11)	1725
Chest	502 (1.98)	2102 (1.66)	2604
Ear, nose, and throat	184 (0.73)	1198 (0.95)	1382
Elbow	361 (1.42)	2096 (1.66)	2457
Eye(s)	377 (1.49)	5443 (4.30)	5820
Internal Organs	203 (0.80)	396 (0.31)	599
Knee	2107 (8.31)	8945 (7.06)	11,052
Lower Arm	510 (2.01)	5159 (4.07)	5669
Lower Back Area	3918 (15.46)	11,692 (9.23)	15,610
Lower Leg	591 (2.33)	3235 (2.56)	3826
Mouth and facial bones	179 (0.71)	1844 (1.46)	2023
Multiple Head Injury	278 (1.10)	2356 (1.86)	2634
Multiple Lower Extremities	296 (1.17)	1101 (0.87)	1397
Multiple Neck Injury	85 (0.34)	478 (0.38)	563
Multiple Trunk	146 (0.58)	637 (0.50)	783
Multiple Upper Extremities	510 (2.01)	2067 (1.63)	2577
Multiple body parts	3654 (14.42)	13,031 (10.29)	16,685
Other	315 (1.24)	1128 (0.89)	1443
Shoulder(s)	1437 (5.67)	5285 (4.17)	6722
Skull	350 (1.38)	2244 (1.77)	2594
Soft Tissue (Head)	273 (1.08)	3126 (2.47)	3399
Soft Tissue (Neck)	236 (0.93)	1016 (0.80)	1252
Spinal Cord	40 (0.16)	115 (0.09)	155
Upper Arm	251 (0.99)	1597 (1.26)	1848
Upper Back Area	443 (1.75)	1816 (1.43)	2259
Upper Leg	228 (0.90)	1380 (1.09)	1608
Vertebral column	718 (2.83)	1867 (1.47)	2585
Wrist and hand	3547 (13.99)	29,640 (23.41)	33,187
Total	25,345	126,614	151,959

**Table 4:** Log-gamma regression models estimating the mean payments and burden associated with specific nature of injury categories for those claims that resulted in a payment, stratified by medical and death and disability claims.

Nature of Injury	Medical Claims		Disability and Death Related Claims	
	Mean Payment ($)	95% CI ($)	Mean Payment ($)	95% CI ($)
Amputation	3849	(1396, 10,608)	3724	(2093, 6626)
Asphyxiation	1488	(648, 3421)	295	(146, 595)
Bite/sting	383	(345, 426)	1252	(535, 2925)
Burn	528	(498, 561)	3129	(2477, 3953)
Contusion	673	(645, 702)	3508	(3059, 4024)
Crushing	697	(627, 774)	3597	(2782, 4652)
Dislocation	1336	(1094, 1631)	5615	(3898, 8087)
Foreign body	470	(446, 496)	2047	(1430, 2928)
Fracture	1307	(1160, 1472)	6111	(5175, 7216)
Hearing loss (traumatic only)	622	(348, 1110)	2930	(1489, 5764)
Heat prostration	1371	(1192, 1576)	2516	(1790, 3537)
Irritation	303	(256, 359)	400	(274, 585)
Laceration	621	(597, 647)	2437	(2142, 2773)
Multiple injuries	924	(864, 987)	4586	(3802, 5533)
Poisoning	572	(484, 675)	2359	(1084, 5136)
Puncture	558	(534, 584)	3253	(2258, 4687)
Rupture	805	(594, 1090)	14,285	(7772, 26,255)
Sprain	830	(798, 863)	4230	(3694, 4844)
Strain	862	(828, 896)	4603	(4177, 5073)
Unknown	471	(396, 561)	2371	(1565, 3591)
Carpal tunnel syndrome	1047	(820, 1337)	4529	(3085, 6648)
Vision loss	542	(422, 694)	1775	(843, 3738)
All other cumulative injuries	963	(758, 1224)	7519	(5483, 10,310)
All other specific injuries, not otherwise classified	737	(689, 789)	3471	(2947, 4088)
Concussion (reference)	1187	(1037, 1359)	3817	(3077, 4735)

**Table 5:** Log-gamma regression models estimating the mean payments and burden associated with specific source of injury categories for those claims that resulted in a payment, stratified by medical and death and disability claims.

Source of Injury	Medical Claims		Disability and Death Related Claims	
	Mean Payment ($)	95% CI ($)	Mean Payment ($)	95% CI ($)
Burn or Scald—Dust, Gases, Fumes or Vapors	884	(567, 1377)	470	(195, 1129)
Burn or Scald—Electrical Current	876	(694, 1107)	3062	(1798, 5217)
Burn or Scald—Fire or Flame	732	(574, 934)	4555	(2905, 7142)
Burn or Scald—Hot Objects or Substances	498	(461, 537)	2539	(1959, 3292)
Burn or Scald—Steam or Hot Fluids	527	(466, 596)	2873	(1917, 4305)
Burn or Scald—Temperature Extremes	961	(806, 1146)	2642	(1771, 3942)
Burn or Scald— not otherwise classified	609	(519, 715)	1744	(1148, 2651)
Caught In/Between— Machine or Machinery	938	(780, 1128)	3202	(2245, 4568)
Caught In/Between—Object Handled	669	(594, 753)	4756	(3239, 6983)
Cut/Puncture/Scrape—Broken Glass	583	(534, 637)	1832	(1378, 2437)
Cut/Puncture/Scrape—Hand Tool, Utensil Not Powered	568	(541, 597)	1756	(1473, 2095)
Cut/Puncture/Scrape—Object Being Lifted or Handled	588	(556, 622)	2374	(1838, 3065)
Cut/Puncture/Scrape—Powered Hand Tool, Appliance	731	(671, 796)	2554	(1861, 3506)
Caught In/Between— not otherwise classified	616	(582, 652)	2466	(1909, 3186)
Cut/Puncture/Scrape— not otherwise classified	572	(546, 598)	3166	(2380, 4211)
Fall/Slip—From Different Level (Elevation)	868	(804, 937)	5183	(4050, 6633)
Fall/Slip—From Ladder or Scaffolding	1068	(847, 1345)	7136	(4950,10,289)
Fall/Slip—From Liquid or Grease Spills	1019	(832, 1249)	5113	(3638, 7186)
Fall/Slip—Into Openings	992	(790, 1247)	4566	(2598, 8024)
Fall/Slip—On Ice or Snow	997	(778, 1277)	3852	(2782, 5332)
Fall/Slip—On Same Level	800	(732, 875)	4888	(4033, 5925)
Fall/Slip—On Stairs	854	(775, 941)	4596	(3082, 6853)
Fall/Slip— not otherwise classified	852	(811, 894)	4899	(4227, 5677)
Miscellaneous Causes	744	(683, 810)	3893	(3313, 4574)
Miscellaneous Causes—Absorption, Ingestion, Inhalation	568	(510, 634)	2280	(1085, 4790)
Miscellaneous Causes—Foreign Matter in Eyes and Ears	434	(411, 459)	1985	(1344, 2932)
Miscellaneous Causes—Person in Act of a Crime	554	(501, 613)	5715	(3809, 8575)
Motor Vehicle—Collision with another Vehicle	945	(849, 1051)	4770	(3258, 6984)
Motor Vehicle—Collision with a Fixed Object	1089	(798, 1486)	6439	(3129, 13,251)
Motor Vehicle—Vehicle Upset	1924	(1120, 3302)	4656	(2739, 7916)
Motor Vehicle—Collision, not otherwise classified	1071	(908, 1264)	4834	(2930, 7974)
Rubbed/Abraded—Repetitive Motion	812	(639, 1032)	2422	(1318, 4448)
Strain/Injury By—Holding or Carrying	944	(803, 1110)	6387	(4474, 9119)
Strain/Injury By—Jumping	978	(596, 1605)	6228	(2958, 13,115)
Strain/Injury By—Lifting	885	(835, 938)	4661	(4069, 5338)
Strain/Injury By—Pushing or Pulling	827	(780, 877)	4740	(3865, 5813)
Strain/Injury By—Reaching	802	(702, 916)	3877	(2961, 5076)
Strain/Injury By— repetitive Motion–Carpal Tunnel Syndrome	1014	(949, 1083)	5020	(4010, 6285)
Strain/Injury By— not otherwise classified	829	(759, 905)	4148	(3572, 4818)
Strain/Injury By—Twisting	797	(739, 860)	3655	(3012, 4437)
Strain/Injury By—Using Tool or Machinery	984	(586, 1652)	4434	(2494, 7882)
Striking/Stepping—Moving Parts of Machine	798	(540, 1180)	3822	(2416, 6048)
Striking/Stepping—Object Being Lifted or Handled	645	(587, 708)	2264	(1805, 2839)
Striking/Stepping—Stationary Object	679	(572, 806)	2555	(2076, 3143)
Striking/Stepping—Stepping on Sharp Object	477	(409, 555)	3414	(1225, 9519)
Striking/Stepping—Striking Against or Stepping on	715	(652, 783)	3397	(2586, 4463)
Struck/Injured By—Another Person	651	(620, 684)	4295	(3398, 5429)
Struck/Injury By—Animal or Insect	531	(492, 574)	2535	(1961, 3276)
Struck/Injury By—Falling or Flying Object	720	(668, 775)	4509	(3498, 5813)
Struck/Injury By—Hand Tool or Machine in Use	727	(635, 832)	3536	(2821, 4433)
Struck/injury by—Motor Vehicle	756	(664, 862)	5536	(3615, 8478)
Struck/Injury By—Moving Parts of Machine	818	(642, 1041)	5468	(2346, 12,742)
Struck/Injury By—Object Being Lifted or Handled	750	(684, 823)	3760	(3104, 4553)
Struck/Injury By—Object Handled by Others	678	(600, 767)	4533	(2889, 7114)
Struck/Injury By— not otherwise classified	634	(598, 672)	3205	(2690, 3817)
Burn or Scald—Chemicals (reference)	468	(410, 534)	1829	(1365, 2450)

**Table 6:** Log-gamma regression models estimating the mean payments and burden associated with specific body part injured for those claims that resulted in a payment, stratified by medical and death and disability claims.

Body Part Injured	Medical Claims		Disability and Death Related Claims	
	Mean Payment ($)	95% CI ($)	Mean Payment ($)	95% CI ($)
Abdomen including Groin	772	(672, 886)	4661	(3565, 6094)
Ankle and Feet	686	(649, 725)	3381	(2959, 3862)
Brain	944	(630, 1415)	7732	(2571, 23,251)
Buttocks and hip	688	(620, 763)	4617	(3463, 6157)
Chest	630	(583, 681)	3233	(2458, 4252)
Ear, nose, and throat	571	(515, 633)	2970	(1924, 4586)
Elbow	793	(706, 891)	3831	(3160, 4644)
Eye(s)	473	(442, 506)	1677	(1284, 2189)
Internal organs	817	(662, 1010)	3451	(1879, 6336)
Knee	824	(761, 891)	4673	(4034, 5414)
Lower arm	618	(582, 656)	3795	(2834, 5084)
Lower back area	843	(804, 884)	3805	(3391, 4270)
Mouth and facial bones	812	(744, 886)	2157	(1651, 2820)
Multiple body parts	934	(890, 981)	6242	(5478, 7112)
Multiple head injury	951	(842, 1074)	2964	(2180, 4030)
Multiple lower extremities	872	(692, 1099)	4266	(2640, 6894)
Multiple neck injury	947	(730, 1229)	4248	(2769, 6515)
Multiple trunk	963	(773, 1200)	4418	(3205, 6090)
Multiple upper extremities	895	(828, 967)	4,161	(3292, 5260)
Other	842	(625, 1133)	3588	(2674, 4814)
Shoulder(s)	863	(806, 923)	6617	(5619, 7793)
Skull	726	(673, 784)	2930	(2325, 3692)
Soft tissue (head)	748	(652, 858)	2737	(2019, 3709)
Soft tissue (neck)	854	(715, 1020)	4659	(2303, 9426)
Spinal cord	911	(722, 1148)	11,115	(2144, 57,637)
Upper arm	630	(560, 708)	4331	(3234, 5801)
Upper back area	759	(697, 827)	3819	(2809, 5192)
Upper leg	670	(553, 812)	2903	(2119, 3977)
Vertebral column	858	(725, 1014)	4232	(3359, 5332)
Wrist and hand	637	(616, 660)	3191	(2825, 3603)
Lower Leg (reference)	732	(617, 868)	3326	(2741, 4037)

## Supplementary Materials

The following are available online at https://www.mdpi.com/1660-4601/17/19/7157/s1. Table S1: Log-gamma regression models estimating the mean payments and burden associated with specific nature of injury categories for those claims that resulted in a payment, stratified by medical and death and disability claims, Table S2: Log-gamma regression models estimating the mean payments and burden associated with specific source of injury categories for those claims that resulted in a payment, stratified by medical and death and disability claims, Table S3: Log-gamma regression models estimating the mean payments and burden associated with specific body part injured for those claims that resulted in a payment, stratified by medical and death and disability claims.
