# Supplementary material for: Analyzing Workers’ Compensation Claims and Payments Made Using Data from a Large Insurance Provider
Source: Int J Environ Res Public Health. 2020 Sep 30;17(19):7157. doi: 10.3390/ijerph17197157 (PMC7579155; doi:10.3390/ijerph17197157)
Supplement: Supplementary file 1 [file ijerph-17-07157-s001.pdf]

Table S1: Log-gamma regression models estimating the mean payments and burden associated with specific nature of injury categories for those claims that resulted in a payment, stratified by medical, and death and disability claims

Nature of injury	Medical claims				Disability and death related claims			
	Mean payment ($)	95% CI ($)	n	Burden ($)	Mean payment ($)	95% CI ($)	n	Burden ($)
Contusion	673	(645, 702)	23,139	**1,557,0157**	3,508	(3,059, 4,024)	3,426	**12,019,173**
Fracture	1,307	(1,160, 1,472)	1,231	1,608,348	6,111	(5,175, 7,216)	732	**4,473,147**
Laceration	621	(597, 647)	13,886	**8,625,843**	2,437	(2,142, 2,773)	1,399	3,409,636
Multiple injuries	924	(864, 987)	3,033	2,802,090	4,586	(3,802, 5,533)	822	**3,770,007**
Puncture	558	(534, 584)	6,522	**3,641,214**	3,253	(2,258, 4,687)	407	1,323,946
Sprain	830	(798, 863)	9,178	**7,618,292**	4,230	(3,694, 4,844)	2,901	**12,271,059**
Strain	862	(828, 896)	29,219	**25,173,196**	4,603	(4,177, 5,073)	9,931	**45,717,300**

The estimates are from GEE models accounting for correlations within claims nested within each client and adjusted for age at injury, gender, years of work experience, employment status, and state

Categories listed as Not Otherwise Classified (NOC) are not included here

Table S2: Log-gamma regression models estimating the mean payments and burden associated with specific source of injury categories for those claims that resulted in a payment, stratified by medical, and death and disability claims

Source of injury	Medical claims				Disability and death related claims			
	Mean payment ($)	95% CI ($)	n	Burden ($)	Mean payment ($)	95% CI ($)	n	Burden ($)
Cut/Puncture/Scrape-Object Being Lifted or Handled	588	(556, 622)	5,782	**3,398,988**	2,374	(1838, 3,065)	266	631,378
Fall/Slip-From Liquid or Grease Spills	1,019	(832, 1,249)	5,353	**5,456,670**	5,113	(3,638, 7,186)	219	1,119,785
Fall/Slip-On Ice or Snow	997	(778, 1,277)	7,815	**7,790,262**	3,852	(2,782, 5,332)	218	839,691
Fall/Slip-On Same Level	800	(732, 875)	1,333	1,066,686	4,888	(4,033, 5,925)	1,065	**5,205,510**
Motor Vehicle-Collision with other Vehicle	945	(849, 1,051)	6,981	**6,593,884**	4,770	(3,258, 6,984)	401	1,912,631
Strain/Injury By-Lifting	885	(835, 938)	2,047	1,811,527	4,661	(4,069, 5,338)	2,475	**11,535,490**

Strain/Injury By-Pushing or Pulling	827	(780, 877)	303	250,611	4,740	(3,865, 5,813)	890	**4,218,485**
Strain/Injury By-Reaching	802	(702, 916)	7,517	**6,026,714**	3,877	(2,961, 5,076)	378	1,465,497
Strain/Injury By- repetitive Motion–Carpal Tunnel Syndrome	1,014	(949, 1,083)	4,249	4,308,958	5,020	(4,010, 6,285)	965	**4,844,689**
Struck/Injured By-Another Person	651	(620, 684)	3,173	2,066,112	4,295	(3,398, 5,429)	1,064	**4,570,357**

The estimates are from GEE models accounting for correlations within claims nested within each client and adjusted for age at injury, gender, years of work experience, employment status, and state
Categories listed as Not Otherwise Classified (NOC) are not included here

Table S3: Log-gamma regression models estimating the mean payments and burden associated with specific body part injured for those claims that resulted in a payment, stratified by medical, and death and disability claims

	Medical claims				Disability and death related claims			
**Body part injured**	**Mean payment ($)**	**95% CI ($)**	**n**	**Burden ($)**	**Mean payment ($)**	**95% CI ($)**	**n**	**Burden ($)**
Ankle and Feet	686	(649, 725)	9,290	**6,369,388**	3,381	(2,959, 3,862)	2,525	8,536,279
Knee	824	(761, 891)	7,253	**5,973,835**	4,673	(4,034, 5,414)	1,999	**9,341,565**
Lower back area	843	(804, 884)	9,674	**8,156,879**	3,805	(3,391, 4,270)	3,740	**14,230,421**
Multiple body parts	934	(890, 981)	10,607	**9,911,186**	6,242	(5,478, 7,112)	3,377	**21,078,582**
Shoulder(s)	863	(806, 923)	4,354	3,757,082	6,617	(5,619, 7,793)	1,366	**9,039,221**
Wrist and hand	637	(616, 660)	24,961	**15,906,040**	3,191	(2,825, 3,603)	3,377	**10,774,844**

The estimates are from GEE models accounting for correlations within claims nested within each client and adjusted for age at injury, gender, years of work experience, employment status, and state
Categories listed as Not Otherwise Classified (NOC) are not included here
